# Beneficial role of naringin against methotrexate-induced injury to rat testes: biochemical and ultrastructural analyses

**DOI:** 10.1080/13510002.2022.2101832

**Published:** 2022-07-21

**Authors:** Hany Elsawy, Abdullah M. Alzahrani, Manal Alfwuaires, Ashraf M. Abdel-Moneim, Mahmoud Khalil

**Affiliations:** a Department of Chemistry, College of Science, King Faisal University, Al-Ahsa, Saudi Arabia; b Department of Chemistry, Faculty of Science, Tanta University, Tanta, Egypt; c Department of Biological Sciences, College of Science, King Faisal University, Al-Ahsa, Saudi Arabia; d Department of Zoology, Faculty of Science, Alexandria University, Alexandria, Egypt; e Department of Biological Sciences, Faculty of Science, Beirut Arab University, Beirut, Lebanon

**Keywords:** Methotrexate, naringin, testicular toxicity, oxidative stress, ultrastructure, antioxidants, testosterone, nitric oxide

## Abstract

**Background:**

Methotrexate (MTX) is a commonly used chemotherapeutic drug that has adverse toxic effects on germ cells. Naringin (NG) is a natural flavanone glycoside, with different phytotherapeutic applications, and its possible protective effects against MTX-induced testicular tissue damage were investigated in this study.

**Methods:**

Low and high doses of NG (40 and 80 mg/kg/day) were given for 10 days by intraperitoneal (i.p.) injection and MTX (20 mg/kg i.p.) was given at the 4th day of the experiment, with or without NG in rats.

**Results:**

The obtained results showed that exposure to MTX increased malondialdehyde (MDA) levels and nitric oxide (NO) production compared with the control. In the meantime, MTX depleted catalse (CAT), superoxide dismutase (SOD), glutathione reductase (GR), glutathione peroxidase (GPx), and reduced glutathione (GSH) in the testicular tissue. Further, serum testosterone levels were significantly decreased in the MTX group. NG significantly counteracted the aforementioned effects of MTX; however, NG80 was more effective in restoring SOD, GR, MDA and NO. Interestingly, NG80 achieved a better improvement in the ultrastructural pattern of the testicular cells in MTX-exposed rats.

**Conclusion:**

These results indicated, for the first time, that NG could be a potential candidate therapy against MTX-reprotoxic impacts.

## Introduction

Methotrexate (MTX) is a potent chemotherapeutic agent used in the treatment of several types of malignancies and inflammatory diseases [1]. MTX is a folic acid analog that suppresses the production of nuclei acid precursors by inhibiting dihydrofolate reductase enzyme [2]. This, in turn, leads to lethal effects on cancer cells and accounts for MTX toxicities to normal tissues that have high proliferation rates such as the hematopoietic cells and the gastrointestinal mucosal cells [3]. Therefore, some restrictions have been made on its clinical applications because of its adverse health consequences [4]. Previously, MTX has been reported to cause seminiferous tubule degeneration, reduction of sperm numbers, and sperm DNA mutations [5,6]. Oxidative stress was documented as a key player in the pathogenesis of MTX-induced testicular damage [7–9]. Excessive generation of reactive oxygen species (ROS) has been linked to atrophy in seminiferous tubules and apoptosis in spermatocytes [10].

In recent literature, natural antioxidants have been used in order to minimize the side effects due to MTX administration [11]. Flavonoids are non-nutritive dietary components that possess diverse biochemical and pharmacological properties [12]. Naringin (4′,5,7-trihydroxy flavonon 7-rhamnoglucoside, NG) is the predominant flavanone found in grape fruit and related citrus species. Grapefruit contains around 17 mg NG/100 g of edible fruit [13]. When the highly lipophilic NG is given orally, it is converted by the intestinal microflora into its absorbable form (i.e. naringenin) [14]. A 400 mg/day dose of NG has been shown to improve the antioxidant system and lipodystrophy in hypercholesterolemic subjects [15]. Several studies have suggested that NG has anticancer, antiviral, anti-inflammatory, hepatoprotective and nephroprotective activities [16–20]. Furthermore, NG can also reduce the mutagenic potential of radiation [21,22].

Earlier study of Butchi Akondi et al. [23] demonstrated that NG improved all sperm parameters in type 1 diabetic rats. Moreover, NG was able to manage reproductive toxicity of bisphenol A through reduction of oxidative stress [24]. Thus, the use of NG may be a feasible approach to counteract MTX-induced testicular damage. The aim of this study is to elucidate the protective effects of NG on the testis of rats exposed to MTX. We explored testicular redox status, testosterone level and ultrastructural aspects of the testis.

## Materials and methods

### Chemicals

Naringin (NG) (Cat. No.: 71162), methotrexate (MTX) (Cat. No.: M9929), and all other reagents used in this study were supplied by Sigma Chemicals Co. USA.

### Animals

Twenty-four male Wistar albino rats, aged 3–4 months (220 ± 30 g body weight) were placed at a regulated environment (12 h light:dark cycles, 25 ± 3°C temperature, and 50 ± 5% relative humidity). Rats were fed with commercial food pellet and water *ad libitum*. Animal handling and experimental procedure were approved by the Ethics committee of King Faisal University, Saudi Arabia (KFU-REC/2019-03-05).

### Experimental design

After acclimatization to laboratory conditions for 2 weeks, male rats were divided randomly into four groups (six per group) as follows:

- Control (vehicle treated): rats received saline daily by intraperitoneal (ip) injection for 10 days
- MTX: rats received a single ip injection of MTX (20 mg/kg b.w.) on the fourth day of the experiment
- MTX + NG40: rats were treated with ip daily dose of NG (40 mg/kg b.w.) for 10 days; on the fourth day, rats received MTX (20 mg/kg b.w.)
- MTX + NG80: rats were treated with ip daily dose of NG (80 mg/kg b.w.) for 10 days; on the fourth day, rats received MTX (20 mg/kg b.w.)

**Figure 1.**
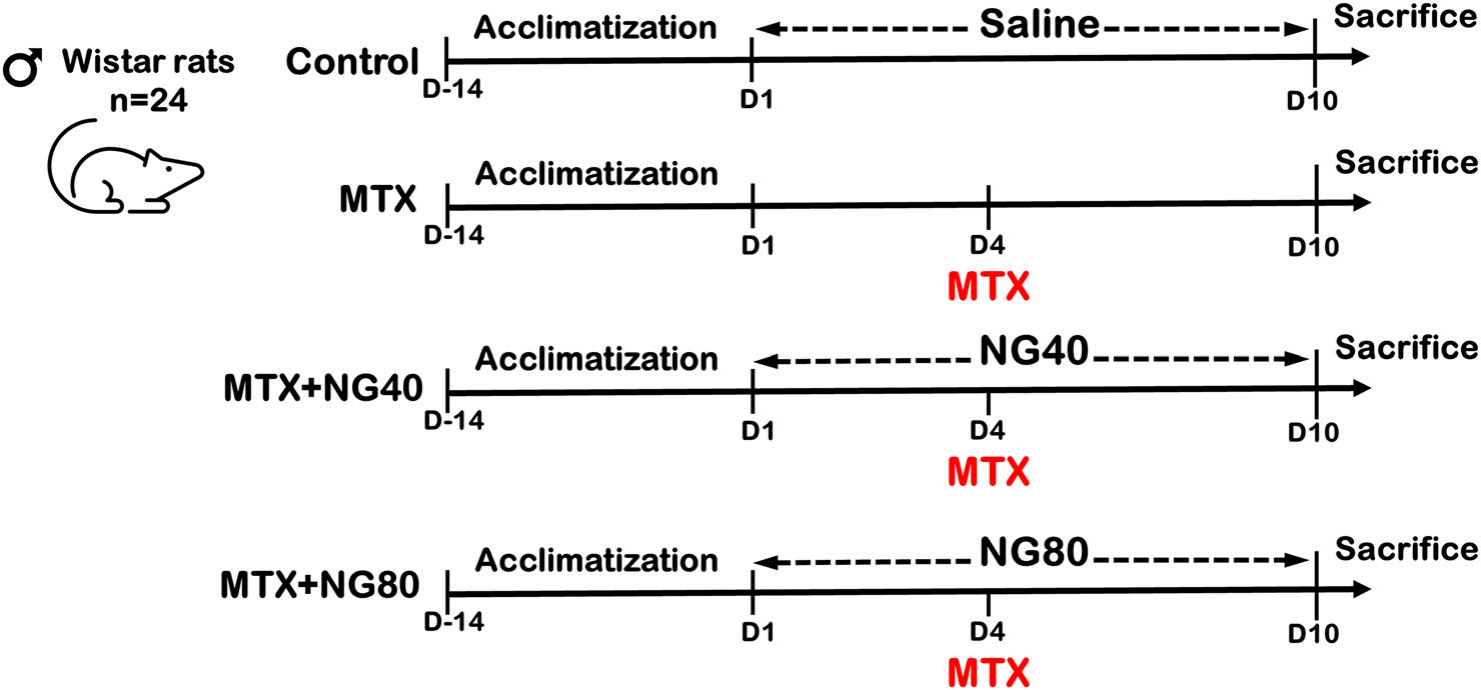
Time schedule of drug treatments.

The experimental procedure is illustrated in Figure 1. The injected MTX dose was previously found to reduce male fertility and was shown to cause testicular cell damage in rats [25]. The doses of NG were selected based on published research suggesting that NG was not toxic at these doses [26]. At the end of experimental period, rats were sacrificed by cervical dislocation under light ether anesthesia. Both testes from each rat were dissected out, washed using chilled saline solution. The left testis was minced and homogenized (10% w/v) in 50 mM ice-cold phosphate buffer saline (pH 7.0) and centrifuged (5000 rpm for 15 min at 4 °C). The resulting clear supernatant was used for the assay of tissue biochemical parameters related to oxidative stress. Sera were obtained from blood samples after centrifugation at 3400 rpm for 20 min, and stored at −80°C for subsequent analysis of testosterone. Small slices of the right testis were cut, cleaned, washed with normal saline, and fixed for transmission electron microscopy.

### Estimation of testis antioxidant parameters

Catalase (CAT), superoxide dismutase (SOD), glutathione peroxidase (GPx), glutathione reductase (GR), and reduced glutathione (GSH) content were assayed according the manufacturer’s guidelines (CAT; CAT No.: CA2517, SOD; CAT No: SD2521, GPx; CAT No.: GP2529, GR; CAT No.: GR2523, GSH; CAT No.: GR2511; Bio-Diagnostic, Egypt). The colorimetric assay of CAT involves the enzymatic decomposition of hydrogen peroxide at 240 nm [27]. The principle of SOD assay depends on the ability of the enzyme to inhibit the phenazine methosulphate-mediated reduction of nitro-blue tetrazolium dye [28]. Measurement of GPx activity is based on an indirect measure of the activity c-GPx. Oxidized glutathione (GSSG), produced upon reduction of an organic peroxide by c-GPx, is recycled to its reduced state by glutathione reductase (GR). This is accompanied by oxidation of NADPH (GR coenzyme) to NADP+ [29]. The assay procedure of GR depends on the reduction of GSSG in the presence of NADPH, which is oxidized to NADPH+ [30]. The determination of GSH content utilizes a carefully optimized enzymatic recycling method, using GR. The thiol of GSH reacts with DTNB and produces a yellow colored 5-thio-2-nitrobenzoic acid (TNB). The mixed disulfide, GSTNB that is concomitantly produced, is reduced by GR to recycle the GSH and produce more TNB. The rate of TNB production is directly proportional to this recycling reaction, which is turn directly proportional to the concentration of GSH in the sample [31]. The total protein contents of the tissue samples was determined using the Bradford method [32].

### Measurement of oxidative markers in the testis

Malondialdehyde (MDA) was determined spectrophotometrically using the Lipid Peroxide Kit (CAT No.: MD2528, Bio-Diagnostic, Egypt) according to the instructions of the supplier. Measurement of MDA levels was based on the reaction of thiobarbituric acid (TBA) with MDA in an acidic medium at 95°C, to form a TBA reactive product [33]. Results were expressed as nmol/g tissue.

Nitric oxide (NO) levels were measured by the method of Griess diazotization reaction using the Nitrite Assay Kit (CAT No.: NO2533, Bio-Diagnostic, Egypt). The values of NO were represented as µmol/g tissue.

### Determination of serum testosterone levels

Testosterone was analyzed from serum using ELISA kit with a reportable range of 0.083–16 ng/ml (Cat. No. EIA1559, DRG International, Marburg, Germany).

### Transmission electron microscopy (TEM)

Testicular samples of 2 mm² were immediately fixed in 3% glutaraldehyde and phosphate buffer saline (PBS; pH = 7.2) at 4 °C for 3 h, before post-fixation in 1% osmium tetroxide (OsO_4_) for 1 h. OsO_4_ was washed away with the same buffer. Specimens were dehydrated through a graded ethanol serious, and embedded in Araldite. Thin sections (80–100 nm) were cut with glass knives on an ultramicrotome (EM UC7, Leica Microsystems), and double-stained on copper grids with saturated uranyl acetate (20 min) and lead citrate (10 min) [34]. Jeol JEM-1011 electron microscope (JEOL, USA) was used for examination and photography of the specimens at 80 kV.

### Statistical analysis

Data were expressed as mean ± standard error (SE) for animals in each group. One-way analysis of variance (ANOVA) and post hoc tests (Turkey) were performed using SPSS, version 10 (SPSS Inc., Chicago, IL, USA) to determine the differences among the groups. Differences were considered significant at values of *p* < 0.05.

## Results

### NG enhances antioxidant defenses in rats treated with MTX

**Figure 2.**
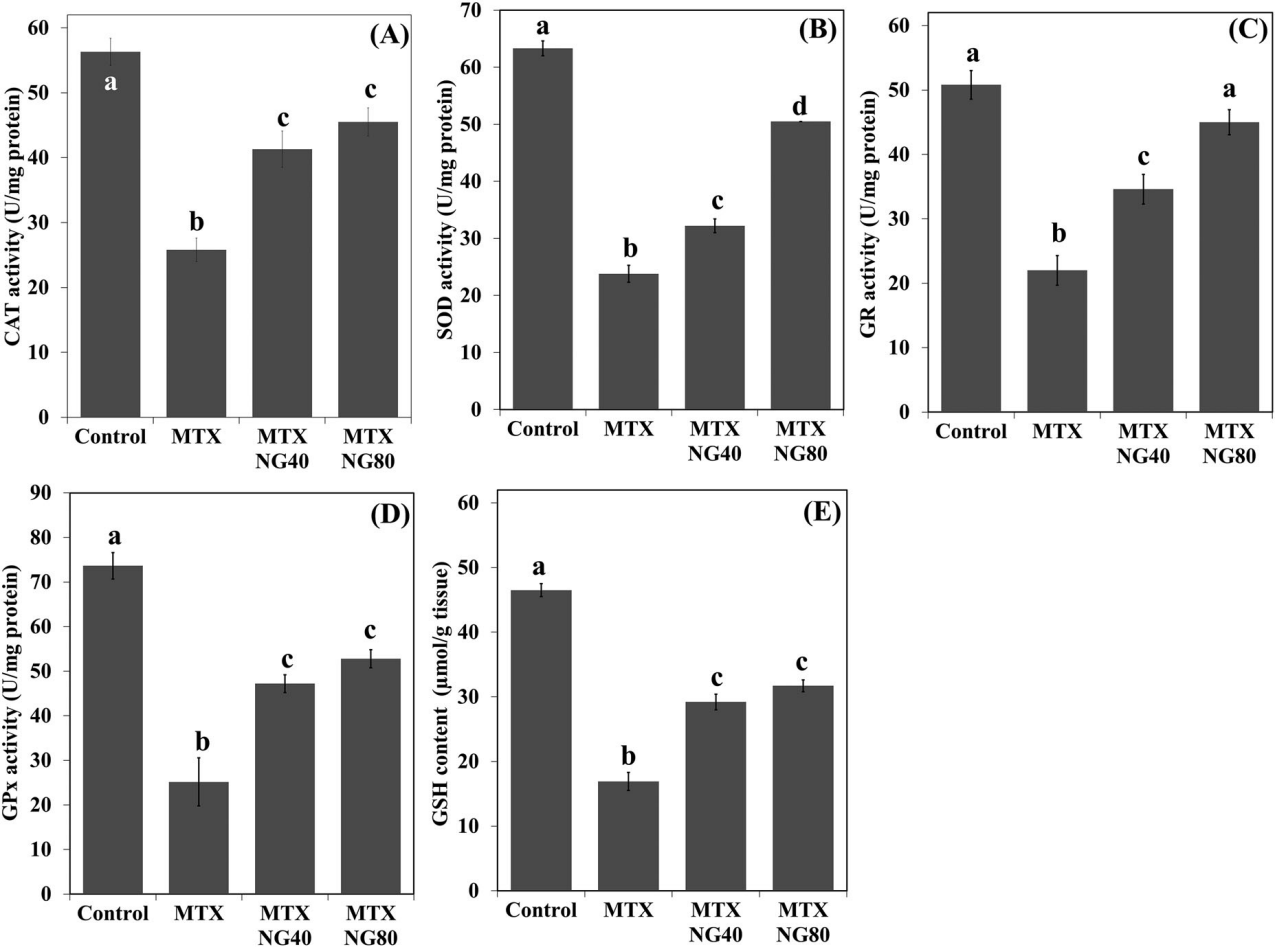
Effect of MTX and/or NG on antioxidant defense system. Each value represents the mean ± SE. Bar chart with different letters indicate significant difference at *p* < 0.05 (One-Way ANOVA).

The results showed that MTX caused a significant decrease in CAT, SOD, GR, GPx and GSH in the testis when compared with the control group (Figure 2(A–E)). Administration of NG40 and NG80 reversed MTX-induced reductions of antioxidant levels when compared with the MTX group; however, NG80 was superior in restoring SOD and GR.

### NG decreases testicular MDA and NO, and upregulates serum testosterone in rats treated with MTX

**Figure 3.**
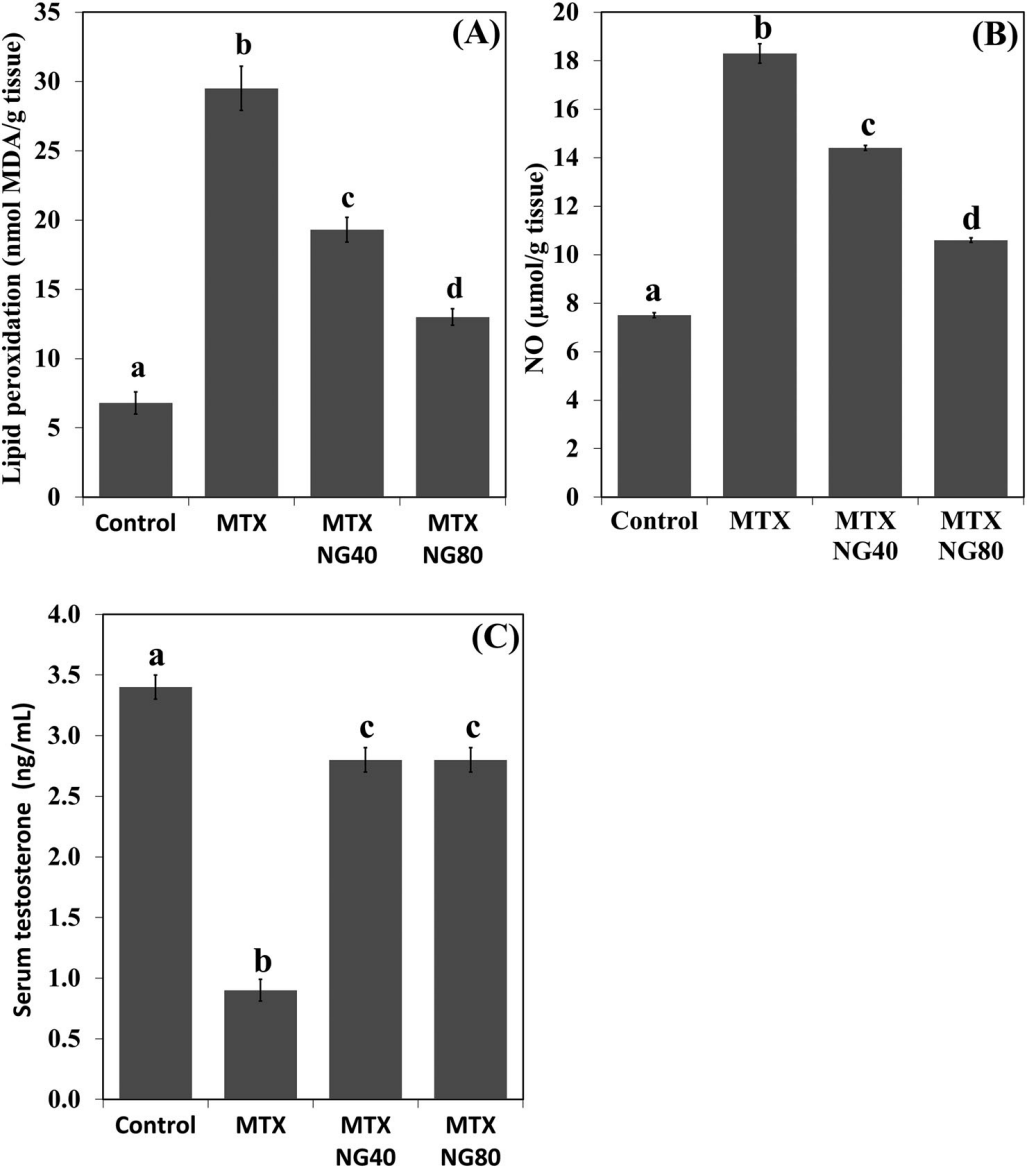
Effect of MTX and/or NG on testis MDA and NO levels, and serum testosterone. Each value represents the mean ± SE. Bar chart with different letters indicate significant difference at *p* < 0.05 (One-Way ANOVA).

Levels of MDA and NO were significantly increased in the rat testis following MTX exposure when compared with the control group (Figure 3(A,B)). NG treatment produced a significant dose-dependent reduction in MDA and NO levels. On the other hand, serum testosterone level was lower in the MTX group than in the control animals (Figure 3(C)). Both NG40 and NG80 could prevent MTX-induced decrease in serum testosterone.

### NG attenuates testicular ultrastructural damage in rats treated with MTX

**Figure 4.**
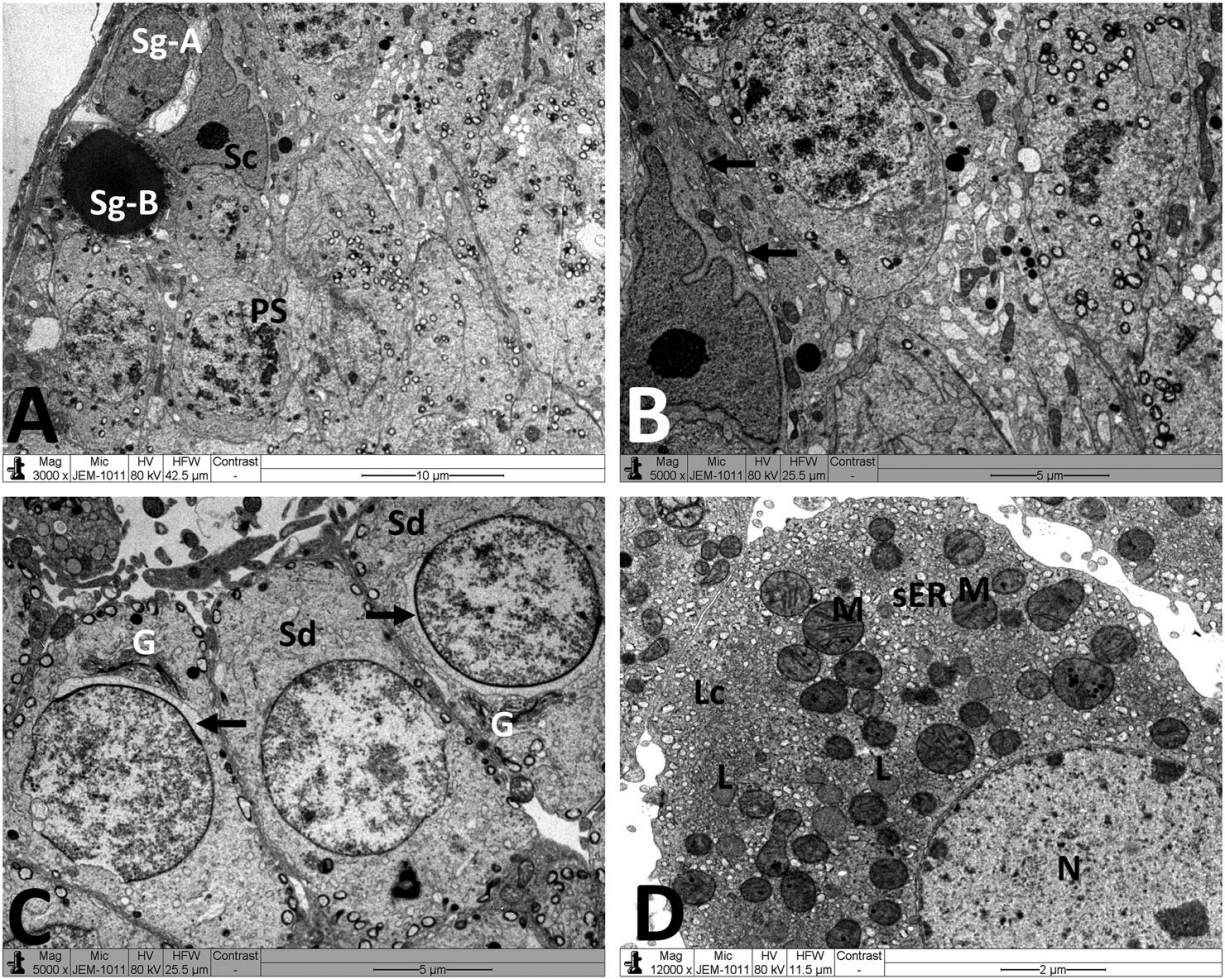
Electron micrographs of testicular tissue from the control group. (A) Normal appearance of spermatogonia type A (Sg-A) and B (Sg-B), primary spermatocytes (PS) and Sertoli cell (Sc). (B) Higher magnification of Figure 3(A) showing Sertoli-Sertoli junctional complexes (arrows). (C) Round spermatids (Sd) at the phase of acrosomal formation. Arrow: acrosomal cap, G: Golgi apparatus. (D) Leydig cell (Lc) with nucleus (N) depicting fine granular chromatin and clear nuclear membranes, mitochondria (M), smooth endoplasmic reticula (sER) and lipid droplets (L). Scale bar: 10 µm (A), 5 µm (B, C), 2 µm (D).

**Figure 5.**
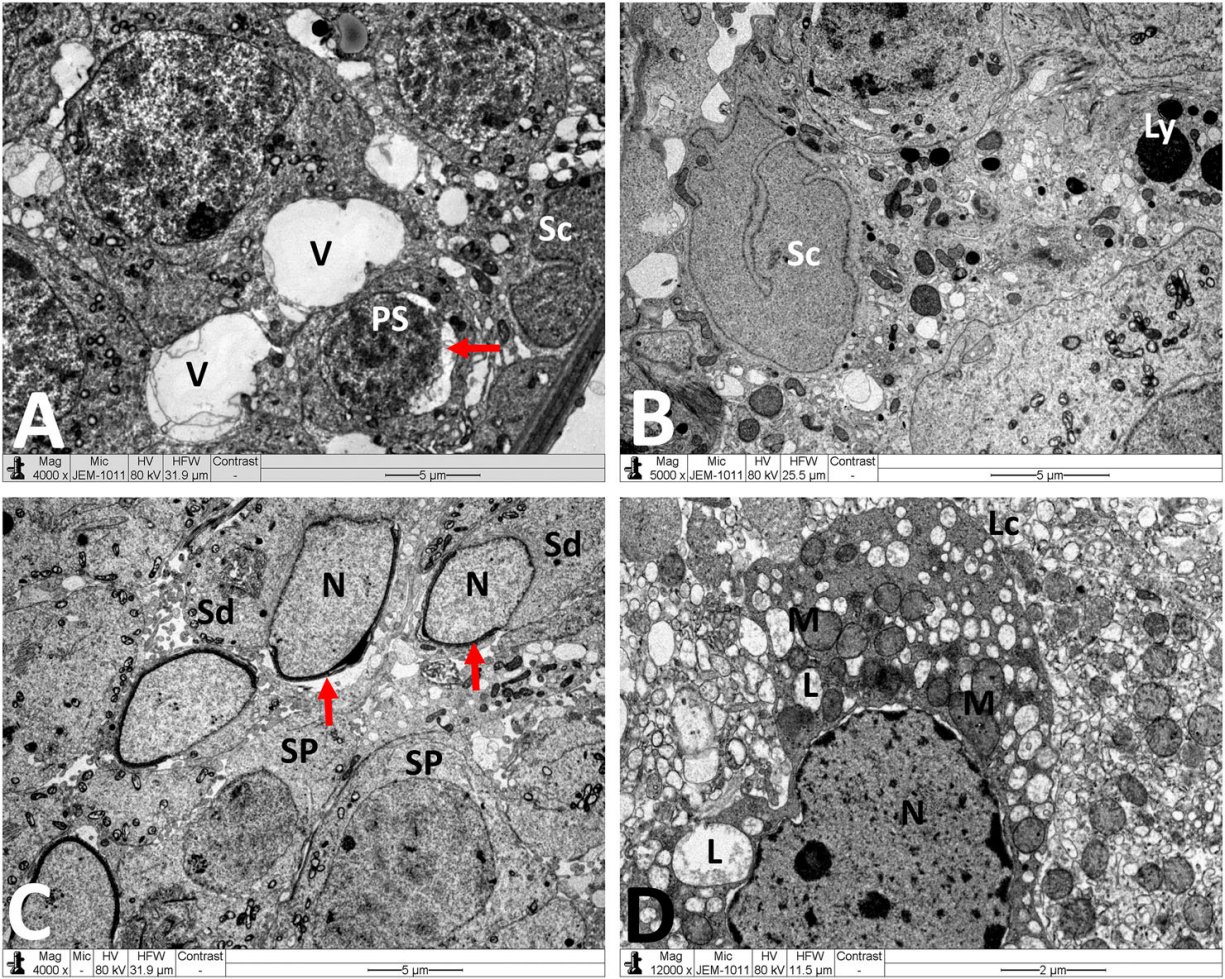
Electron micrographs of testicular tissue from the MTX group. (A) Part of Sertoli cell (Sc) with extensive cytoplasmic loss and vacuolation (V). Note also: deformed primary spermatocyte (PS) displaying perinuclear space (arrow). (B) Sertoli cell (Sc) showing a large number of primary and secondary lysosomes (Ly). (C) Early stage spermatid (Sd) appears with marked irregularities of the nucleus (N) and acrosomal cap (arrow). SP: primary spermatocyte. (D) Deleterious changes in Leydig cell (Lc) are discernible. The nucleus (N) shows contended peripheral chromatin and distended envelope. In the cytoplasm, observe the presence of many lipid vacuoles (L), sparse smooth endoplasmic reticula, and disintegrated mitochondria (M) with broken cristae. Scale bar: 5 µm (A–C), 2 µm (D).

**Figure 6.**
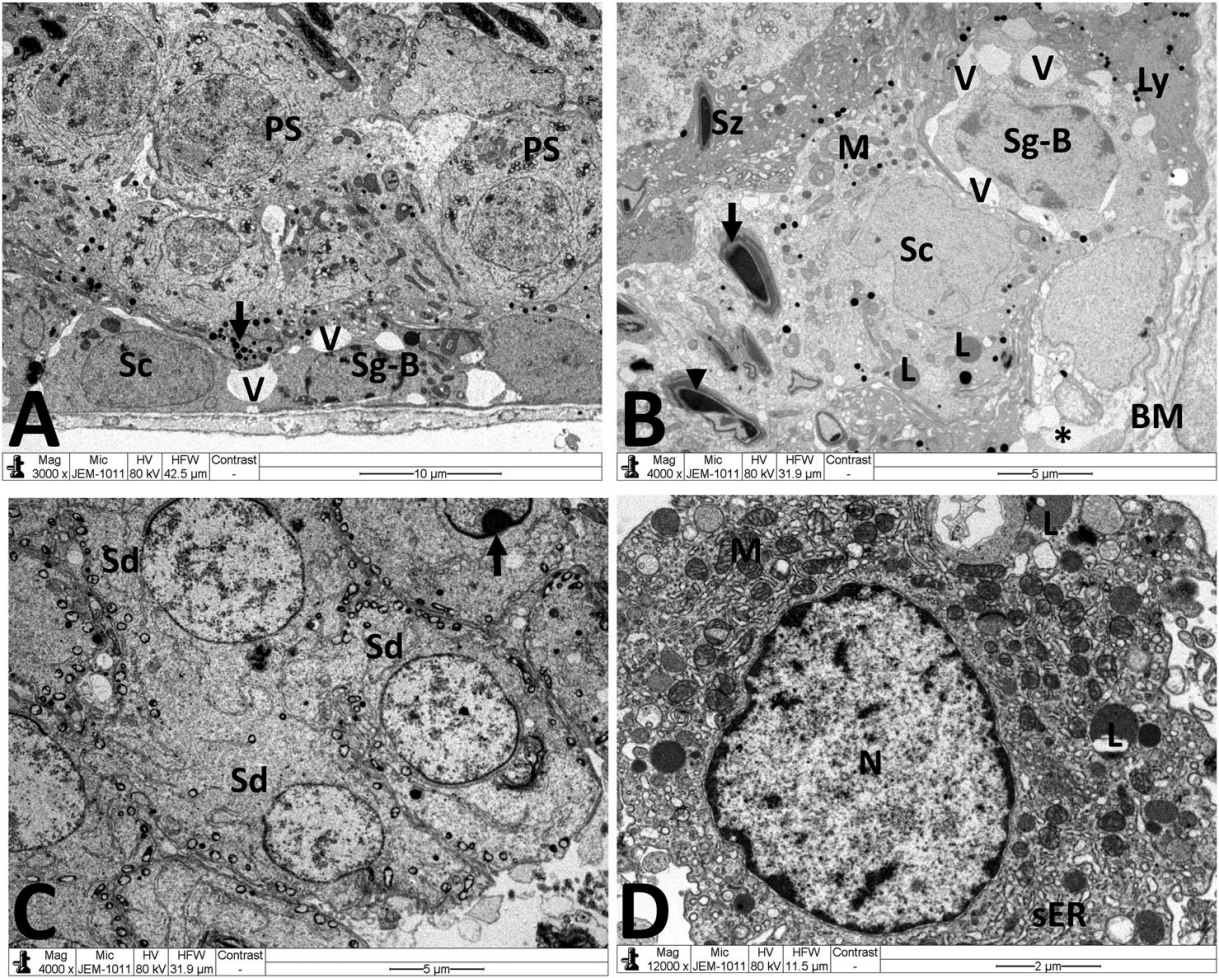
Electron micrographs of testicular tissue from the MTX + NG40 group. (A) Sertoli cell (Sc), lysosomes (arrow) in Sertoli cell cytoplasm, spermatogonium type B (Sg-B), vacuoles (V) between spermatogonia and Sertoli cell, and primary spermatocytes (PS) in normal ultrastructure. (B) Marked detachment (*) of Sertoli cell (Sc) from basal lamina (BM), vacuoles separation areas (V) between spermatogonia type B (Sg-B) and Sertoli cell (Sc), M: mitochondria, L: lipid inclusions, Ly: lysosomes, spermatozoon (Sz) in normal ultrastructure, and degenerated spermatozoa with loss of acrosome (arrow) and nuclear fragmentation (arrowhead). (C) Normal ultrastructure features of early spermatids (Sd) at different developmental stages. Arrow points to abnormal dislocation of acrosomal vesicle inside spermatid nucleus. (D) Leydig cell nucleus (N) with less condensed chromatin, and normal mitochondria (M), minimal lipid inclusions (L) and prominent smooth endoplasmic reticula (sER) in Leydig cell cytoplasm. Scale bar: 10 µm (A), 5 µm (B, C), 2 µm (D).

**Figure 7.**
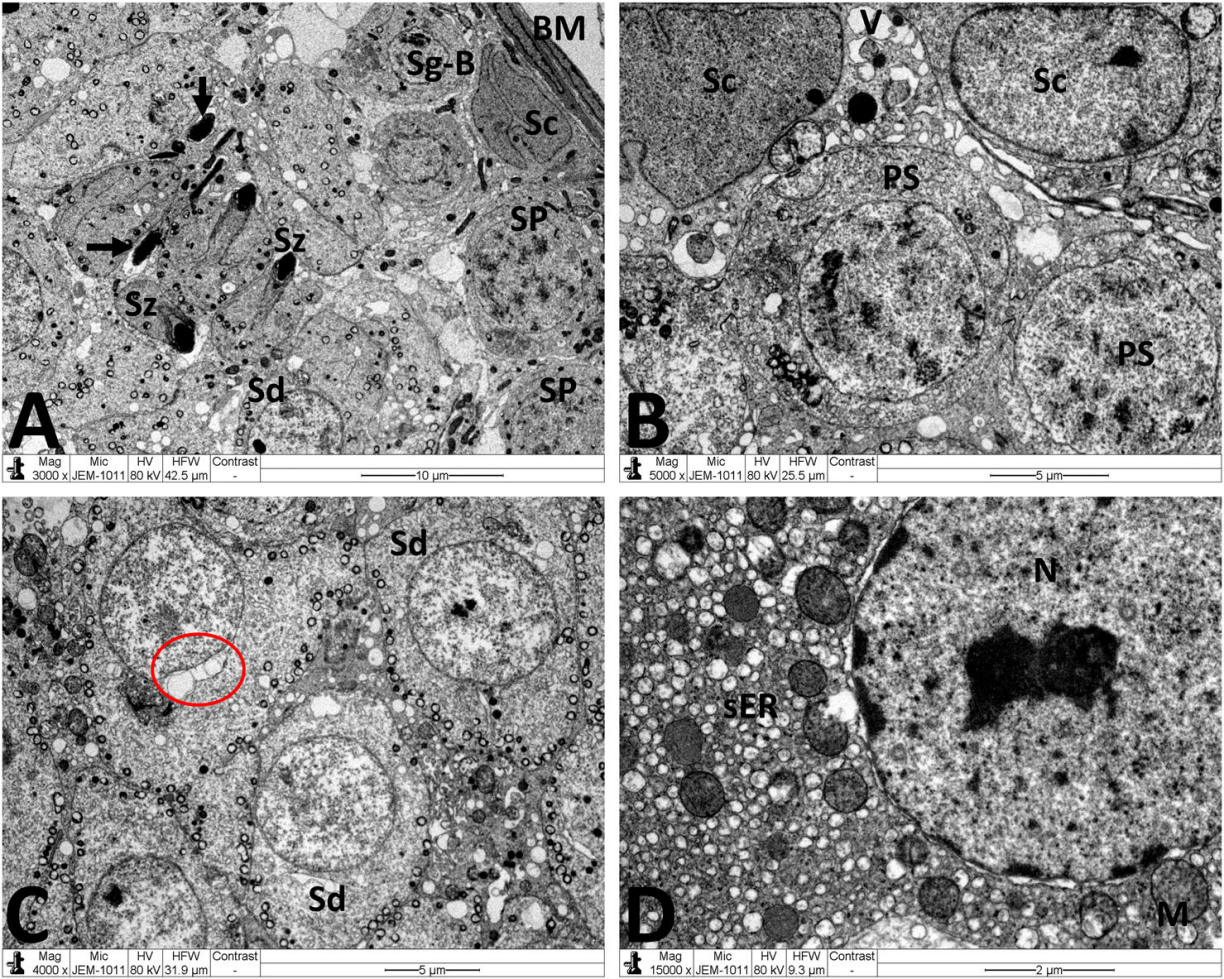
Electron micrographs of testicular tissue from the MTX + NG80 group. (A) Spermatogonia type B (Sg-B) and Sertoli cell (Sc) are located on basal lamina (MB), spermatocytes (SP), early spermatids (Sd) and spermatozoa (Sz) are noticed similar to the control group, and fragments of damaged spermatozoa (arrows) can also be seen. (B) Sertoli cells (Sc) with small empty spaces and vacuoles (V), and well-preserved contact between Sertoli cells and primary spermatocytes (PS). (C) Normal morphology of early spermatids (Sd). Observe malformed spermatid with vacuolated acrosomal region (red circle). (D) Normal Leydig cell nucleus (N), mitochondria (M), and smooth endoplasmic reticula (sER). Scale bar: 10 µm (A), 5 µm (B, C), 2 µm (D).

Normal testicular ultrastructure was observed in control rats. The seminiferous tubules had regular basement and were lined by two types of spermatogonia: types A and B (Figure 4(A)). Type A spermatogonia had large ovoid and lightly stained nuclei. Type B spermatogonia had smaller and spherical nuclei with a more electron dense nucleoplasm. In primary spermatocytes, the nuclei had faint chromatin accumulation and spherical mitochondria at the periphery. Sertoli cells extending radially from the basement membrane to the lumen of the tubule and were identified by their large pale nuclei and numerous mitochondria. Tight junctional complexes between Sertoli cell processes separated spermatogonia from primary spermatocytes (Figure 4(B)). In the adluminal compartment of seminiferous tubules, early stages of spermiogenesis were noted as the formation of acrosomes over the anterior hemisphere of the spermatid nucleus to form an apical acrosomal cap (Figure 4(C)). Normal Leydig cells were identified near blood capillaries. These cells had a large euchromatic nucleus, numerous mitochondria, abundant smooth endoplasmic reticulum (sER) and few lipid droplets (Figure 4(D)). In the MTX group, Sertoli cells exhibited severe cytoplasmic vacuolation. Some of these vacuoles separated Sertoli cells from the neighboring germ cells and/or basement membrane (Figure 5(A)). Intensive accumulation of primary and secondary electron-dense lysosomal elements were observed in other Sertoli cells (Figure 5(B)). In addition, electron micrographs showed degenerative changes in the whole of the cell lineage of spermatogenic epithelium. Spermatogonia and spermatocytes showed shrunken nuclei and perinuclear wide spaces. Spermatids were the most affected cells and most of them had oddly shaped nuclei with abnormal acrosomal capping and vacuolation of the cytoplasm (Figure 5(C)). In the interstitium, the nuclei of Leydig cells showed condensed-clumped nuclear chromatin at the periphery and dilated envelope. Moreover, their cytoplasm contained damaged mitochondria, less-developed sER and large number of lipid vacuoles (Figure 5(D)). Electron micrographs of the testis of animals received MTX + NG40 showed morphologically normal spermatogonia and primary spermatocytes (Figure 6(A)). However, varying degrees of separations and vacuole structures were noted in the connection regions between spermatogonium-basement membrane, spermatogonium-Sertoli cell, and spermatogonia. Lysosomal accumulation was still seen in the Sertoli cells. Regarding spermatids and spermatozoa, some were intact, and others were distorted (Figure 6(B,C)). Leydig cells were comparable to those of the control group (Figure 6(D)). MTX + NG80 group showed better restoration of spermatogenesis toward the normal architecture (Figure 7(A–D)).

## Discussion

Chemotherapeutic agents could disturb physiological homeostasis in different organs leading to harmful side effects in non-tumor cells by free radical generation, along with oxidant damage [35]. Previous studies revealed that, MTX could lead to major testicular toxicity [6,8,36]. In this study, we have demonstrated for the first time that NG could counteract MTX-induced testicular lesions in male rats. It has been reported that MTX exposure directly damages Leydig cells [37]. From the electron microscopic results, the presence of numerous lipid vacuoles in MTX-treated testes could reflect the impaired Leydig cell secretory function and the abnormal accumulation of steroid precursors. Moreover, the mitochondrial damage observed by electron microscopy of Leydig cells may also contribute to the decline of testosterone secretions by these cells. In fact, actively respiring mitochondria are essential for LH-induced Leydig cell steroidogenesis [38]. In this regard, Heidari et al. [39] showed that MTX toxicity elicits mitochondrial dysfunction, respiratory chain defects and reduction of intracellular ATP synthesis. Another pathway, which could be involved in suppression of testosterone, is evident by increased testicular pro-oxidant MDA and NO levels together with decreased antioxidant defense [40]. A well-characterised consequence of stress-induced stimulation of the testicular NO release is the decrease in steroidogenic and antioxidant enzyme activities [41]. All this suggests that the germ cell degenerative changes observed in this study might be due to hormonal depletion. On the other hand, our results confirmed that NG significantly increased the plasma testosterone levels in MTX-challenged rats. This may be attributed to NG antioxidant and free radical scavenger activities. Indeed, several reports have demonstrated that the use of antioxidant-based therapies can break down the oxidative chain reactions and play a very significant role in increasing the body’s capacity to fight free radical damage and therefore improve the process of spermatogenesis [42].

Free radicals and lipid peroxidation (LPO) are two leading factors in MTX-induced testicular pathology [36]. LPO takes place in the unsaturated lipids and is involved in the formation of active oxygen [43]. MDA is the end product of LPO and its level is widely used as an index of oxidative stress. In particular, testis is highly vulnerable to oxidative damage due to its high content of polyunsaturated fatty acid [44]. Evidence has revealed that NO overproduction could stimulate tissue injury, in addition to oxidative stress, through interactions with superoxide to form peroxynitrite, a potent cytotoxic agent [45], and cause altered spermatogenesis and spermatic failure [46]. After NG treatment, there was a significant reduction in MDA levels in testicular tissues, which is essential for sperm quality and function. Our results are consistent with previous work [23].

A rise in MDA levels depicts antioxidant failure and results in cellular toxicity. Reducing SOD activity results in the accumulation of superoxide anion, which in turn inhibits CAT enzyme [47]. Reducing CAT activity decreases the ability of the testicles to eliminate H_2_O_2_ produced after exposure to MTX, and this may aggravate the induction of oxidative damage to lipids, proteins and DNA and disable the antioxidant mechanisms [48]. In addition, the GPx may act directly as an antioxidant enzyme, which is involved in inhibition of sperm lipid peroxides and H_2_O_2_ [49]. The low GPx level in MTX-exposed rats can lead to an increase in H_2_O_2_ production or a decrease in GSH concentration [50]. The GSH redox cycle is recognized as an important intracellular antioxidant system. GSH has a major contribution to the maintenance of cell integrity because of its involvement in cell metabolism and reducing properties. In addition to working as a direct free-radical scavenger, GSH also functions as a substrate for GPx and Glutathione-S-transferase (GST). GST plays a critical role in defending the organism against reactive electrophiles by removing them through conjugation with GSH [51]. In addition to the functions of GSH itself, the GSH/GSSG redox couple acts to maintain the redox environment of the cell. In this study, NG as a polyphenol could combat ROS produced by MTX and reverse the decrease in antioxidant enzymes like CAT, SOD and GPx, as well as non-enzymatic GSH levels. Our results are in harmony to those reported with other natural antioxidants in MTX testicular toxicity [52,53]. In another in vitro model of cardioprotection, NG has been proved to induce the phosphorylation of ERK1/2, PKCδ, and AKT, which subsequently activate Nrf2 and its downstream antioxidant genes [54].

In the MTX group, we noticed numerous intercellular gaps in-between germ cells and/or Sertoli cells lining the seminiferous tubules. These findings illustrate a break in cell–cell connections and loss of integrity of blood testis barrier (BTB), and suggest that actin filament bundles, which are the hallmark ultrastructure of the BTB, are a primary target of MTX. Perturbations of BTB are associated with abnormal spermatogenesis, accumulation of damaged/ unhealthy mitochondria and leads to infertility. NG80 was found to alleviate disorders in cellular contact and ultrastructural features of BTB damage in the testis. Recent papers reported that antioxidant supplementation could improve the expression of Sertoli cell junction proteins in murine testis through reduction of ROS burden [55–57].

**Figure 8.**
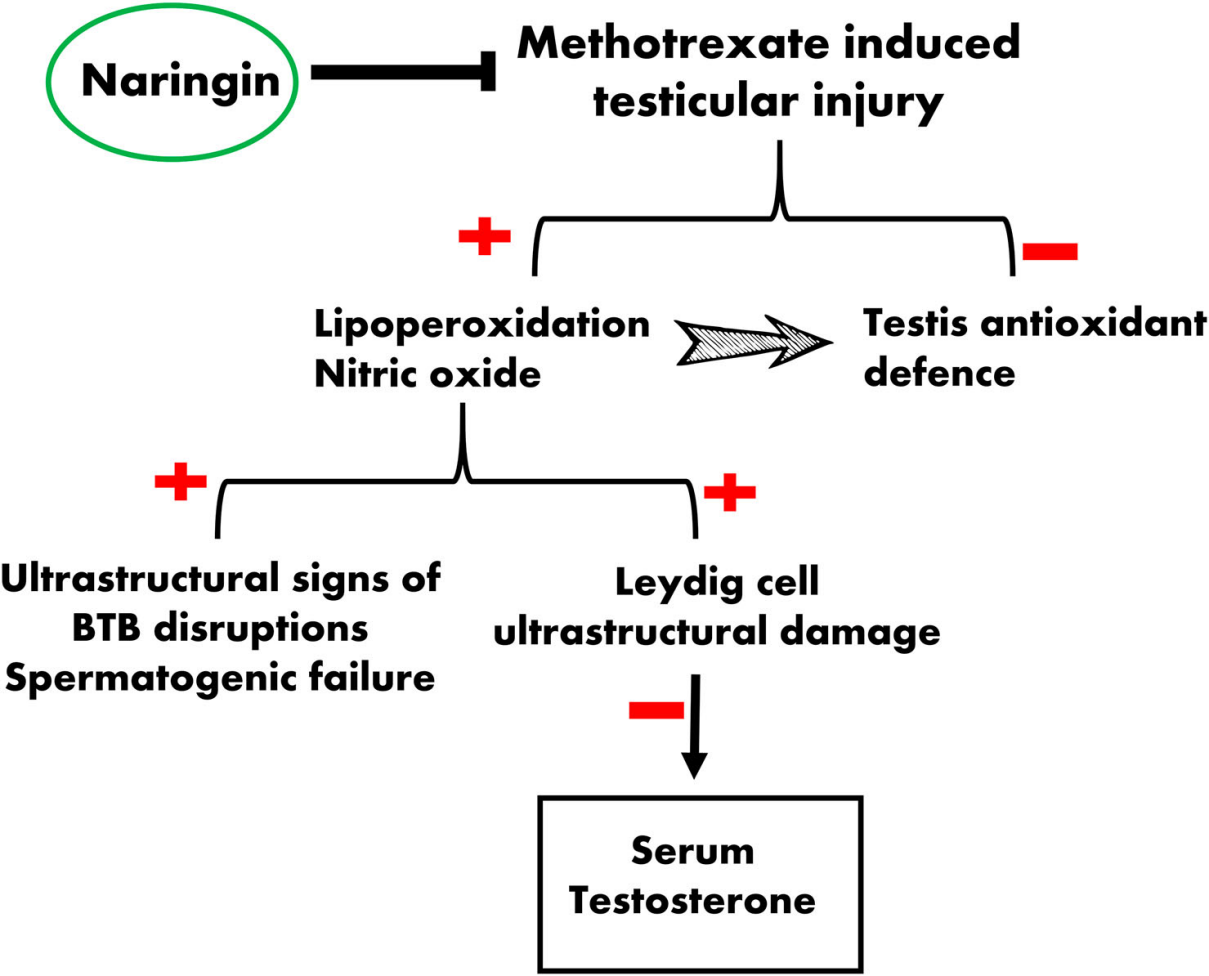
Schematic diagram illustrating the protective potential of NG during MTX-induced testicular oxidative stress.

In conclusion, our study reveals that NG restored MTX-induced testis damage by decreasing MDA and nitrogen species generation and by increasing the levels of endogenous antioxidants. NG has the potency to relieve MTX-induced subcellular pathology especially in Leydig (testosterone producing) cells and BTB (Figure 8). Therefore, NG may serve as potential supplementation, enhancing reproductive health in males undergoing MTX chemotherapy. More detailed studies using immunohistochemistry and/or RT–PCR are needed to clarify the underlying molecular mechanism(s) and validate our findings.

## Data Availability

The authors declare that data supporting the findings of this study are available within the article.
